# Characterising the performance measurement and management system in the primary health care systems of Malawi

**DOI:** 10.4102/phcfm.v16i1.4007

**Published:** 2024-01-31

**Authors:** Martha K. Makwero, Tony Majo, Praveen Devarsetty, Manushi Sharma, Bob Mash, Luckson Dullie, Wolfgang Munar

**Affiliations:** 1 Department of Family Medicine, Faculty of Medicine, Kamuzu University of Health Sciences, Blantyre, Malawi; 2 Department of Primary Health Care Research, Faculty of Medicine, George Institute for Global Health, Telengana, India; 3 Division of Family Medicine and Primary Care, Faculty of Public Health, Stellenbosch University, Cape Town, South Africa; 4 Department of Global Health, Milken Institute School of Public Health, George Washington University, Washington DC, United States of America

**Keywords:** primary health care, performance measurement, performance management, data, goals

## Abstract

**Background:**

Performance Measurement and Management (PMM) systems are levers that support key management functions in health care systems. Just like many low- and middle-income countries (LMICs), Malawi strives to improve performance via evidence-based decision making and a suitable performance culture.

**Aim:**

This study sought to describe PMM practices at all levels of primary health care (PHC) in Malawi.

**Setting:**

This study targeted three levels of PHC, namely the district health centres (DHCs), the zones, and the ministry headquarters.

**Methods:**

This was a qualitative exploratory research study where decision-makers at each level of PHC were engaged using key-informant interviews (KII) and focus group discussions (FGDs).

**Results:**

We found that there is a weak link among levels of PHC in supporting PMM practices leading to poor dissemination of priorities and goals. There is also failure to appropriately institute good PMM practices, and the use of performance information was found to be limited among decision-makers.

**Conclusion:**

Though PMM is acknowledged to be key in supporting health service delivery systems, Malawi’s PHC system has not fully embarked on making this a priority. Some challenges include unsupportive culture and inadequate capacity for PMM.

**Contribution:**

This study contributes to the understanding of the PMM processes in Malawi and further highlights the salient challenges in the use of information for performance management. While the presence of policies on PMM is acknowledged, implementation studies that deal with challenges are urgent and imperative.

## Background

Primary health care (PHC) was given new impetus by the inter-governmental Declaration of Astana in 2018 and subsequent operational plans from the World Health Organization (WHO).^1^ The WHO sees PHC as having three key components: multi-sectoral policy and action, empowered communities, and integrated primary care services with essential public health functions.

The WHO recently published a new framework to guide the monitoring of health systems through the lens of PHC.^1^ This framework conceptualised PHC in terms of a logic model, where health system determinants support service delivery, which determines the attainment of health system objectives. Each of these three domains, namely, multi-sectoral policy and action, empowered people and communities, and primary care and essential public health functions is split into sub-domains, and indicators are suggested for how countries can monitor the entire system. Monitoring these domains is seen as monitoring capacity (health system determinants), performance (service delivery), and impact (health system objectives).

Performance Measurement and Management (PMM) is therefore, a prerequisite for improving health systems performance and requires a focus on service delivery.^2^ The purpose for performance information is twofold. Firstly, to serve as accountability tool, where in democratic era, citizens are involved in public affairs and hold the right to access information. Secondly, performance information is used by decision-makers and healthcare providers as an input in the process of improving service delivery and, ultimately, patient-level and population health outcomes.^3^ In this framework, service delivery is divided into essential processes and outputs. Processes include the model of care, systems for improving the quality of care as well as resilience of health facilities. Outcomes include access and availability of services as well as quality of services. The quality of services is related to the core primary care functions (first contact accessibility, continuity, coordination, comprehensiveness, and person-centredness) and patient safety, effectiveness, efficiency, and timely access to care. The implication is that any PMM system should systematically monitor all these essential components of service delivery.

Performance Measurement and Management encompasses systems for governance and management of health systems and services. It facilitates decision-making for planning, monitoring and evaluation, and quality improvement (QI) monitoring, and improving performance within health institutions. It is a continuous set of routine processes that monitor progress towards achieving performance targets and informs QI initiatives to address identified gaps. Further, these systems should have feedback loops, to give their results back to the end users of the service rendered. In addition, these processes should be part of a comprehensive and continuous system of QI.

Comprehensive and accurate health data availability is a critical component of performance monitoring cycles and managers ought to be capable of using this information to diagnose problems, and identify and implement solutions.^4^ Several interventions have therefore been put in place to strengthen the capacity of managers to engage in more consultative and evidence-based cycles of measuring, planning, implementing, evaluating, and adjusting solutions to improve performance.^5^ Many low- and middle-income countries (LMICs) have established Health Management Information Systems (HMIS) to enhance routine health centre (HC)-based data management.^5^ Various countries have embarked on strengthening the quality, relevance, and comprehensiveness of their data as a step towards enhancing informed decision-making and QI.^6^ Implementation of the PMM process requires organisational commitment and a paradigm shift in the internal mechanisms of the use of routine data.^7^ This therefore calls for careful study and modification of the determinants and drivers of PMM, such as organisational values and attitudes.

Malawi is among the low-income countries in Southern Africa, hoping to strengthen the implementation of its HMIS, through enhancement of data collection and use at all levels of the health system. The 2015 HMIS policy for Malawi is also a means of responding to global protocols, such as Sustainable Development Goals (SDGs) and Universal Health Coverage (UHC).^8^ Malawi’s 2017–2022 Health Sector Strategic Plan (HSSP) has also emphasised the need for quality health information that is accessible to all intended users. Such information should enable performance assessment and evidence-based decision-making across all programmes. To achieve this, the plan proposed: (1) the need for comprehensive knowledge management in the health sector, and (2) the continuous building of a harmonised and coordinated National Health Information System (NHIS) under a Central Monitoring and Evaluation Department (CMED). Furthermore, the Malawi government intends to strengthen the public sector HMIS through consensus building and alignment with the Christian Health Association of Malawi (CHAM), the second biggest health service provider, and with private healthcare facilities.

Although there are some documented efforts and successes in PMM, there remains limited use of routine data and information.^5^ The 2015 Health Information System (HIS) policy assumed that the tools and processes for data management processes are limited.^8^ Thus, it further highlighted that there is deficiency in the use of performance information by decision-makers and their experiences in PMM is largely unknown. There is the need therefore to understand how the PMM is currently working and what issues need to be addressed to improve the system. This study therefore aimed to characterise the processes of PMM in the PHC context of Malawi and highlight the challenges in order to inform areas of improvements.

## Methodology

### Study design and setting

This was a descriptive exploratory qualitative study design. In Malawi, PHC is delivered through the Essential Health Package (EHP) Programme. The programme refers to a prioritised, but a selective package of basic and cost-effective promotive, preventive, curative, and rehabilitative health services. These are determined based on scientific and practical experience in service delivery and their ability to have an impact on health status.^9^ There are PHC key packages of services in Malawi. There are water and sanitation, food and nutrition, immunisation against major childhood diseases, prevention and control of locally endemic diseases, treatment for common diseases, and provision of essential drugs.^10^ Each package is directly linked to a specific programme within the Ministry of Health (MOH). The programmes include preventive, promotion, curative and rehabilitative service tracks.

Malawi’s health system is organised into four levels, namely, community, primary, secondary and tertiary, which are connected via an established referral system. Community, primary and secondary levels of care fall under the health districts. Malawi’s health system is divided into five health zones: South West, South East, Central West, Central East, and the North. Each health zone is responsible for looking after specific districts and linking them to the ministry’s headquarters. The zones offer a technical quality management (QM) and PMM function to the districts under them. The function of the central MOH includes policy making, standard setting, quality assurance, strategic planning, resource mobilisation, technical support, monitoring and evaluation, and international representation.

### Conceptual framework

There are various models of performance management (PM) proposed by various authors with each model designed to address the specific PM needs. Through the lens of Newton-Lewis et al.^4^ LMICs have deficiency PMM approaches, a thing believed to reduce organisational outcomes. The authors have hence proposed the suitable model for LMICs.

**FIGURE 1:**
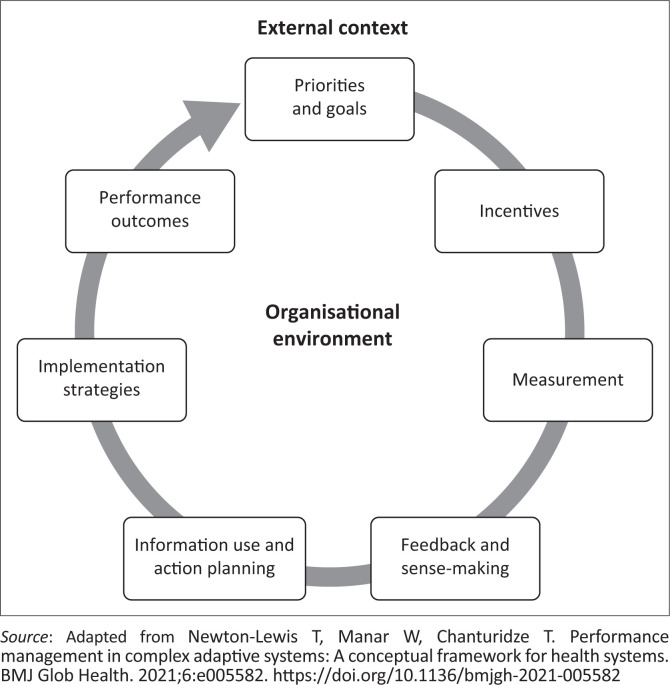
The Performance Measurement and Management conceptual framework.

This study was hence informed by a conceptual framework by Newton-Lewis et al.,^4^ WHO monitoring and evaluation framework, and the HIS policy for Malawi. These three are considered relevant to the study as they are all well aligned with the study’s main objectives. Newton-Lewis framework, for example, highlights the interrelationship of several components of PMM as shown in Figure 1. The framework is preferred as it highlights the continuity of all the key processes in PMM as a closed circuit. Through the lens of Newton-Lewis framework, each component has been described as follows:

1. **Performance goals and priorities:** A highly performing PHC organisation should achieve the desired goals and priorities. Goals and priorities are regarded as the first component of the PMM cycle and are considered the reason for the organisation existence. They give direction and influence how other components within the PMM cycle are designed.
2. **Implementation support:** Implementation support, on the other hand, is defined as the tactics (practices and routines) used by organisations to improve performance that is aligned with the goals and priorities. Implementation support includes reward systems, resourcing, and capacity building, among others. Implementation support ensures that organisational goals are met efficiently and effectively.
3. **Performance measurement:** We defined this as both the processes used to measure performance and defining what is measured. This informs organisations on how they are performing. This helps determine whether the implementation support tactics are effective, or whether there is a need to re-design them. Performance measurement encompasses all processes of data collection, aggregation, analysis, sense-making, and use.
4. **Performance outcomes:** We see these as processes and service improvements that come from the performance measurement cycle.

### Study population and sampling

The study population consisted of managers who were responsible for PMM at different levels of the system. These levels included: the primary care facilities, the district management team, programme coordinators at the health zone, and the national coordinators at the MOH.

In each of the five health zones, we randomly selected one district and in each of those districts we purposively selected three HCs, making a total of 15. From each of the 15 HCs, we purposively selected two decision-makers: the facility in charge and the maternity department in charge. These were selected because they make decisions and lead in the implementation of PMM tasks at the HC level. Maternity department was purposively selected because it is one of the highly prioritised departments within the facility because one of the goals of HSSP is to minimise the maternal mortality rate. Decision-makers needed to be in the health sector for at least 2 years and in their present position for at least 6 months because we thought such work experience made them conversant enough with the PMM processes within the system

At the district level, we interviewed the District Medical Officer (DMO) or District Health Officer (DHO). District Medical Officer or District Health Officer are key decision-makers in the delivery of PHC services at district level. We interviewed two programme coordinators at zonal level. We varied the portfolios of the programme coordinators to capture insights of PMM on different programmes. At the MOH, we interviewed the manager responsible for the quality, planning, and data directorates because of their oversight of the PMM and strategic direction in PHC. Overall, there were 51 respondents including those in the pilot study. Data from each respondent at every level of PHC were collected through in-depth interviews (IDIs). There were two FGDs involving respondents from the South West and Central East zones who were randomly identified to participate in the feedback session. Each FGD had 12 participants. The FGDs involved the same participants who were engaged in the IDIs. The FGDs were meant to clarify some issues raised during the IDI sessions, hence the justification for using the same participants.^11^

### Data collection tools and procedures

The study sought approval from College of Medicine Research Ethics Committee (COMREC) before the commencement of data collection activities. Data were collected through the use of semi-structured interview guide developed through the lens of HMIS policy for Malawi, the WHO framework for monitoring and evaluation, and Newton-Lewis framework for PMM. An initial interview with the ex-minister of health also helped to contextualise the interview guide for decision-makers. Opting of an ex-minister for piloting was highly convenient unlike the current one who is usually busy to schedule for a meeting. The other reason was that the ex-minister is conversant with PMM issues and could freely express his experiences on the topic unlike the current minister whom we thought might be conservative for political reasons. To improve the quality of data collection, all research assistants were trained in qualitative interviewing. We employed the research assistants who were not directly connected to the topic, those who were not engaged in any PMM tasks within the field of health or having any connections with the respondents. This helped to minimise the risks of bringing their preconceived notions into the study. The interview guide was piloted to refine the questions for conceptual relevance and clarity. The authors categorised the participants’ social demographic details such as average age, sex, years of experience, and the level of education, see Table 1.

**TABLE 1:** Participants’ social demographic characteristics.

Variable	Health centre level	Districtlevel	Zonallevel	National level	Total
Age in years (average)	44.1	46.6	51.4	57.8	NA
**Sex**					
Male	18	03	08	02	31
Female	12	02	02	00	14
Years of experience (average)	08	05	16	25	NA
**Level of education**					
Certificate	12	00	00	00	12
Diploma	08	00	00	00	08
Degree	00	05	08	02	18

### Data management and analysis

The study adopted five steps to analyse the data. The steps were: transcription, familiarisation of data coding, categorisation, theme generation, and interpretation.^12^ We analysed data deductively in relation to the themes in the tool. Deductive analysis focused on the three frameworks where the codes were developed from. On the other hand, we adopted an inductive analysis since there were some emergent themes deemed significant to the study but outside the propositions we initially developed. In this study, we organised the data by comparing the interview guides with the responses. This assisted us to pick out the concepts and themes. We adopted both deductive and inductive approach to data analysis; the deductive coding was driven by Newton-Lewis PMM framework, the WHO framework for monitoring and evaluation, and the HIS policy for Malawi. Triangulation of the data collected was achieved by collecting the data from participants at all levels of PHC system: HC, district, zone and the ministry headquarters. For quality assurance purposes, two initial randomly sampled transcripts were coded by four independent coders qualified in qualitative research designs who were sourced within the Kamuzu University of Health Sciences, but outside the department conducting this study. The identified differences in coding were discussed with a larger group of the research team and resolved and updated in the codebook.^11^ NVivo version 12 was used to analyse the data.

The study team included two qualified medical professionals with a lot of interest in PHC. They have done several qualitative studies on PHC in Malawi under the Kamuzu University of Health Sciences, but not specifically on PMM. In this study, their main role was focusing on providing technical assistance to the other members of the team. In order to prevent the two from bringing their feelings and reactions, the study adopted a concept of bracketing.^13^ For instance, the study engaged an expert in qualitative designs who we sourced from the field of education for coding of all the sampled transcripts. This was done to prevent the study team from bringing their undue preconceived notions and prejudices into the study.^14^ The expert also played a key role in summarisation of the study findings. The qualitative research assistants were trained on qualitative data collection before the actual fieldwork. None of the research assistants were either connected to the participants professionally or qualified in either health-related areas or information technology.

## Findings

The themes identified participants’ practical experiences on the four components of the PMM conceptual framework: (1) presence and awareness of priorities and goals, (2) implementation support, (3) performance measurement, and (4) performance outcomes.

### Theme 1: Presence and awareness of priorities and goals for PHC in Malawi

No respondent at the facility and district level was able to mention all eight key priority areas as highlighted in the EHP. Respondents from the periphery of the health system had less knowledge of the priorities and goals formulated at the MOH level. When asked to mention the priorities and goals of MOH, majority of the respondents appeared to just be making assumptions about them. We concluded that most respondents had a vague sense of the priority areas but lacked detail and confidence.

At the PHC level, majority of respondents attributed lack of awareness of the priorities and goals to poor dissemination of policy documents from the MOH headquarters. However, respondents at the MOH attributed the problem to a lack of interest by the players at the bottom levels compounded by negative attitude and the systems’ lack of resources to orient every stakeholder:

‘HIS policy, you know what happens? Let me tell you one thing. When you go actually to the facilities, you ask them, do you have this? They will tell you they don’t have why? They know that if they say yes, they have, and you will ask them further questions. I will give you an example. We had, I moved to every district to do the dissemination of HIS policy, what is in the policy, what is expected of this and all those kinds of things and leaving a copy of this in the districts, if you go to them today, they will tell you they don’t have. Why? When they received it they took it and pushed it into the drawer. When they were removing the furniture that went away together with the furniture. And in most cases, these people don’t read the documents you give them.’ (IDI: Male, Participant 2: MOH level, policy maker)

The awareness of key goals and priorities also varied between the different health programmes. Health care workers (HCWs) from the well-funded programmes such as malaria and antiretroviral therapy (ART) were more knowledgeable about priorities and goals formulated at the ministry level. This increased awareness was because of more frequent contact between programme coordinators and primary care providers. This meant that providers received more supervision and feedback on performance concerning the programmes’ targets. It appeared that this feedback motivated better performance and led to more in-service training. Health care workers see this as not only a form of capacity building, but also a financial incentive because of the training allowances they get. This has made HCWs in such departments to be more capable and motivated. Through increased contacts with their coordinators, majority of HCWs acknowledged to be conversant with the set targets, as such the sense-making of performance information has been reported to be a key in evaluation of performance outcomes:

‘You can understand that we are also talking about funding gap … so only those programs that are well funded, if you go in all the facilities and ask either the in charges or nurses about malaria indicators they will explain clearly because they are engaged in it frequently … so all indicators about malaria they will explain because it’s a well-funded program … they can conduct review meetings every quarter and able to do performance measurement activities … similarly you ask them on issues to do with ART, there is no health center which can fail to explain the indicators of ART because it’s one of the programs which are well funded, but most of the programs are not funded, no wonder indicators are not known since they are not acting as a result only the coordinator knows.’ (FGD: Male, Participant 3: District Level, clinician)

### Theme 2: Implementation support for PHC in Malawi

Sub-themes under implementation support include: (1) Performance culture; (2) Performance rewards, negative sanctions, and accountability structures; and (3) Supervision and feedback; (4) human and technical resources.

#### Sub-theme 2.1: Performance culture

This sub-theme focuses on the organisational processes that appraise and respond to individual performance. Decision-makers at the PHC level did not see themselves as responsible for individual performance appraisal and management. They rather saw themselves as responsible for the workforce as a collective unit and delivering on technical tasks. This meant that there was little individual accountability for poor performance thus, little or no consequences follow.

#### Sub-theme 2.2: Performance rewards, negative sanctions, and accountability structures

Decision-makers at the PHC level felt they had no authority to discipline staff or institute sanctions for poor performance. When poor performance is reported to higher levels, there is no action taken. As a result, facility managers have little motivation to report or deal with poor performance. The study finds that there are gaps within the system that nurture the workers’ values and ethical codes of conduct. It is found that despite the rules and regulations, there is poor enforcement of the same leading to insubordination:

‘They can even challenge [] that you are not my employer … so when you report them to the DHO’s office, they usually do nothing and because of that other employees become demotivated … because of lack of punishments, it becomes difficult to handle such people. There was a need to discipline such people, unfortunately, those mandated to institute disciplinary measures do nothing.’ (FGD, Male, Participant 5: District Level, nurse)

The study further finds that majority of HCWs particularly at HC level complained of deficiency of rewards available to the good performers within the MOH as compared to the other government departments. At the HC level, it was found that they are not provided with financial resources as all the medical supplies come from the DHO. This has made it difficult for some managers to institute measures for motivating the workers. Despite this, few managers mention to heavily rely on extrinsic motivational strategies to reward good performance. For example, they recognise good performance publicly or delegate power in the facility to better performers. The study also finds that there is widespread belief by HCWs particularly at the HC and district level that most promotions were related to political or tribal connections and cronyism. However, a good number of respondents see motivation of any type as key in achieving the performance indicators:

‘I think promotion is a big problem, especially on HCW, you may find that for instance, our colleagues in the police are way ahead of you in terms of a grade even though you were employed within the same time.’ (IDI: Male, Participant 4: District Level, clinician)

‘We are being appraised where we do good, they have to appraise us, be it in any way yea verbally whether they present anything new to us its ok, and even those underground doing the job have to be motivated because the motivated nurse will make sure that all the indicators are good, because all those worrisome indicators will not be there, the worrisome indicators will be strictly monitored when somebody is motivated.’ (IDI: Male, Participant 4: District Level, clinician)

Overall, we find that the system lacks adequate accountability for performance. We have also established that incidences such as negative feedback from the community and exposure in the media trigger a sense of accountability for the facility or individual performance.

Although there is an intention to decentralise power and decision-making in the health system, this does not yet seem to have been operationalised in the health sector. Thus, PM was not viewed as a constructive, supportive or collaborative process, but rather as a top-down hierarchical activity, driven by fear of authority, and supported by control of financial and human resources.

#### Sub-theme 3.3: Inadequate supervision and feedback

Sources of performance feedback for facilities included District Health Management Team (DHMT), funders or various programme communities, programme coordinators, and media. The study found that performance feedback is given to HCWs in different ways. Firstly, through the DHMT supervisory team that come to assess performance and also give feedback. Some respondents complained that such visits are perceived as irregular and unsupportive, others view them as positive towards performance. Secondly, through in-service training where policy makers use it to communicate and fill identified deficiencies in knowledge, skills, and attitudes that are responsible for poor performance:

‘We can get feedback in different forms others in terms of mentorship. This means that programs start from the MOH like malaria, HIV, and AIDS they came to mentor people in terms of performance in how they can improve the indicators. They can also organize training when they have observed that there are gaps in service delivery. Sometimes it comes in form of financial partner aid.’ (IDI: Female, Participant 8: District Level, clinician)

Review meetings at the HC level and district level offer the needed feedback on performance. A few respondents reported feedback during mentorship by members of the DHMT or programme coordinators. Though the majority of respondents were of the view that supportive supervision is a key component of PM, they did not approve the way they are supervised in other departments. For instance, others reported to be usually supervised only when they have failed on their job and not otherwise, and that the supervision is usually authoritative:

‘I think what makes people perform is supervision. If the supervisors come and give feedback on how we are performing, and do supportive supervision. Other supervisors do not come for supervision, they just come and shout at providers so you find that performance goes down. Programs like ART [*Antiret roviral treatment*], they come every quarter, they tell you how you have performed and they give certificates on performance, this motivates workers … this is one of the good drivers … usually when you see a supervisor, it mostly means someone has failed on his or her job … otherwise it’s rare to see a supervisor coming to you and say thank you for a job well done … on[*e*] wonders there is a business as usual culture because there is no appraisal.’ (FGD: Male, Participant 4: District Level, clinician)

Further, feedback can be indirectly given as targeted funding thus funders are often directed towards facilities that are poorly performing. In addition, the media publicity may draw attention to individuals or facilities that are performing poorly.

#### Sub-theme 3.4: Insufficient human and technical resources

The entire process of performance measurement rests in the hands of data officers. They are usually engaged in collecting, capturing, aggregating, and sending data to the next level. However, it was found that most facilities had no qualified data officers. As such, data were being managed by service providers, and support staff such as security officers and ground workers. It was reported that service providers are usually too busy to give the task of data collection the attention it deserves. On the other hand, the support staffs lack the capacity in data collection assignments. All these have been marked as sources of poor performance information because of, among other things, missing of data:

‘I think one of the challenges is HR issue. Sometimes we are unable to collect all the data that we need, I will give you an example. At our non-communicable disease clinics, we do not have a data clerk, so we rely on a nurse or clinician to be the one filling in the registers as well. So, it depends on how busy the clinic is, and how tired these people are, otherwise we end up missing some people from being recorded in the register. What that means, in the end, is that it will affect the data that go into the DHIS. Yes so sometimes we miss people, so inadequate HR affects us.’ (IDI: Female, Participant 10: HC Level, nurse)

Though it is known that the capacity of data collectors is deficient, little effort intended to correct the situation has been observed by a good number of respondents. For instance, it was reported that the ministry lacks financial resources to organise in-service training to capacitate the HCWs on the areas of PM:

‘We told the DHO people, but maybe because of funds, they said they can’t manage. We tried once asking them that they train our data clerks here but they said they will do mentorship. To our surprise, they just mentor only the one they formerly trained.’ (IDI: Male, Participant 12: HC Level, nurse)

Further, the shortage of human resources, particularly qualified data officers, was compounded by high turnover. Though it was not clear as what is causing high turnover, very few respondents particularly at the MOH level opined that it may be because of poor retention strategy, such as poor deployment and rewarding system:

‘But the main challenge that I have seen is that we don’t have enough human resources because when I just came in, I realized that in 2010 the ministry requested the statistical office to recruit 520 data officers. These were posted in data facilities. When I came in 2014, I did the verification and I found out that some of them stopped working. So, they were only about 217 that were remaining in the facilities. And then that means there was a gap from 520 to 217. It was 300 something who had left, it was either for greener pastures or some had just decided to stop because they were sent to hard-to-reach facilities, all these whatsoever. Then I started saying if you want me to provide the information then you need to support me with more people.’ (IDI: Male, Participant 13: Ministry Headquarters, planner)

Generally, decision-makers reported little training in their pre-service and on-job training in health informatics. Respondents at MOH headquarters mentioned though that there are plans to address this problem through the inclusion of PMM aspects on the pre-training programmes. The unavailability of and incapability of HCWs to use technology was another important aspect hindering quality data generation and transmission. The study found that most departments, except for ART, collect data manually through the use of paper-based registers. Tablets or computers were only used for sending reports and not for collecting data, and if these were not available then a paper-based report was sent. Often there were problems with purchasing internet bundles or accessing the network.

### Theme 3: Performance measurement

Sub-themes under performance measurement include (1) data collection processes, and (2) sense-making and use of data.

#### Sub-theme 3.1: Data collection processes

The themes express what kind and how data are collected and utilised for PMM. Further, it tackles how data are analysed, reported, and interpreted to inform PMM. Malawi has a data collection policy HIS and a robust data collection structure. Some sources of information reported to inform performance include: social media audits such as maternal audits, programmatic reviews, community scorecards, informal community feedback as well as supervisor visits.

At the district level, once the data from facilities are aggregated and analysed, final reports are disseminated in the following ways: (1) to the primary care facilities; (2) to funders and other strategic partners; (3) using review meetings; and (4) using bulletins. Different streams of data are religiously transmitted to the central levels by a certain date of the month.

#### Sub-theme 3.2: Sense-making and use of data

While most respondents narrated some level of sense-making and using performance information, others acknowledged that it was a challenge. They admitted to hardly having any time to reflect on the routine data they collect. Stating that decisions pertaining to performance were often based on assumptions, speculation, or experiential information.

It would appear that there is a lack of interest and capacity among decision-makers to routinely make sense of the data they collect unless if it is part of more formal requirements such as review meetings. Again, one respondent alluded that pre-service medical training was purely clinical and did not prepare them for data analytical skills:

‘Okay, I think for me I don’t do it routinely but I do it when there is a need I think all the DH managers get opportunities to appreciate the data in the DHIS through several avenues, one of them is the DIP review meetings that districts conduct because when you are doing the DIP review meetings, the data you use is the data in the DHIS because we understand that not everyone has time and opportunity to look at data and use it to make decisions. So that opportunity arises so it arises regularly.’ (IDI: Male, Participant 9: District Level, DHO)

There was a belief that politicians used their powers to influence decision-making processes and were usually biased towards sections of people that support them. This was seen as a hindrance to evidence-based decision-making by some respondents:

‘At one point we received ambulances and as a health centre we needed to make decisions to say which facilities do these ambulances go, but eventually we looked at the data in terms of how many people are in this catchment area, how many seek care at this health centre on daily basis e.g. how many women give birth at this health centre, so all these things we looked at, we had some guidance to say this is how we will make decisions, this is where each of these ambulances should go. But we got in political influence so that caused a few challenges, so politicians when they were making the decisions they did not look at the data.’ (IDI: Male, Participant 9: District Level, DHO)

Despite this, majority of HCWs at the HC level see data as an instrument supporting technical processes such as the ordering of medication. There were also those few appreciated data as key in PMM:

‘Decisions are made based on the performance on our targets and overall indicators. If the indicators are poor in one area as long as that area is very critical it means decisions should be aimed at improving that set of indicators. Data forms decisions in terms of planning for how to conduct activities like immunization campaigns, sensitization campaigns all these are based on the indicators that we have.’ (IDI: Female, Participant 8: HC Level, nurse)

### Theme 4: Performance outcomes

Most respondents particularly at district and health facility level had little to say about how PMM has been used to improve service delivery processes and outcomes. On probing, a few respondents mentioned examples such as evaluating actual performance, monitoring trends in performance, benchmarking performance against norms and standards, identifying gaps in service delivery, assessing client satisfaction, and planning changes:

‘Like antenatal data for the past months, we have been observing the trend of women starting antenatal care after 12 weeks; it has been up and down. Then we find out that we had a big problem of shortage of pregnant test kit and this has directly affected on a day to day work.’ (IDI, Female, Participant 18: HC level, nurse)

‘All right, so Aaah, for us I will take you at the angle of … a quality improvement or QI yeah, so for QI, we feel like data is very helpful in understanding how the system is performing, okay, if we get data and ask for HMIS officer we look into DHA’s tool and look at the trend of … maybe new-born mortality or outcome, it helps us to understand which district are failing well, which districts are failing badly. And then we also look at what interventions are they lacking, okay, so there it helps us also when it comes to you know, you have, I mean available resources, how do we allocate these resources.’ (IDI: Female, Participant 4: Zonal Level, coordinator)

There were a few practical examples of actions taken as a result of performance information. For example, these included establishing a community scorecard to measure client satisfaction, increasing the number of village clinics to improve access to care, task shifting, and use of support staff to improve Malaria rapid diagnostic testing (MRDT), and timely access to care through audit and feedback, providing in-service training.

## Summary of findings

**FIGURE 2:**
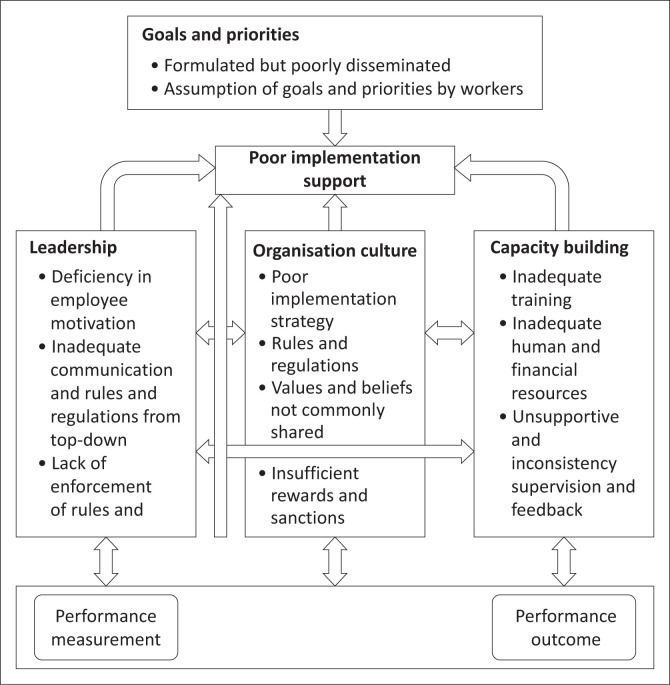
Conceptual Framework for Performance Measurement and Management in Malawi.

We find that the link among the four key aspects of PMM falls short of the recommended WHO framework and Newton-Lewis PMM frameworks.^15^ There are challenges associated with the PMM cycle as narrated by the players in the PMM cycle. For instance, it is commendable that the priorities and goals within the PHC are formulated and disseminated in order to standardising the service delivery and conforming to HSSP, SDGs and other international protocols. However poor dissemination of the same, inadequate pre- and in-service training has resulted in the lack of a unified understanding of the set targets by majority of HCWs. Further, though efforts have been made by MOH to recruit more HCWs and increase the provision of financial resources, the findings show that resources are still deficient thereby thwarting the PMM processes and outputs. We have also found that the system has adopted various means for enhancement of performance such as supervision and feedback mechanism, for example, the use of DHMT to provide quality assurance at the district level. For instance, though supervision and feedback are used as performance tools within the PHC system, they were seen as unsupportive and inconsistent by majority of respondents. The poor incentive system within the PHC is incapable of achieving meaningful performance. The character of PMM practices for Malawi is in contrast with the PMM concept model for Newton-Lewis, which advocates for clear formulation and dissemination of priorities and goals, alignment of priorities and goals to the implementation strategies such as the use of rewards and negative sanctions, capacity building strategies (e.g. training, performance feedback and evaluation), the information processing and use, and performance outcome. Overall, we have summarised the experience of PMM model for Malawi through the conceptual framework (Figure 2).

## Discussion

Whereas the Malawi Growth Development Strategy (MGDs), HSSP, and other documents have highlighted the priorities and goals for Malawi, the lack of awareness of the same is because of a number of factors. Firstly, the strategy for disseminating policy documents which usually contain priorities and goals is not explicitly known by all stakeholders. Mostly, the stakeholders who are engaged put less effort to sensitise their colleagues. Organisational goals are key in realising the needed outcomes. It is further a source of motivation if mechanism are in place to sensitise all players of strategic direction, so that it can be owned and be able to define performance standards.^13^ In this regard, the absence of decision-makers’ understanding and diminished ownership of priorities and goals as in the case of PHC health workers in Malawi might result in a lack of uniformity and coordination among workers since the processes and practices are not harmonised.

Secondly, is need for organisational systems and processes such as PMM organisational culture and strategic objectives to be harmonised to align them with any reform processes.^16^ Priorities and policies are key for attaining better PMM for PHC. It is hence imperative that leaders and managers should be engaged in not only formulating the priorities and goals, but also facilitating its dissemination process. This can enhance the appropriate production and use of performance information leading to improved outcomes.^17^ Appropriate leadership, on the other hand, provides a legitimate impetus in communicating and influencing the acceptance and adoption of the priorities and goals, vision and mission of an organisation.^18^ In this regard, leaders ought to ensure existence and proper utilisation of communication channels.^19^ It is therefore vital that PHC leaders at all levels of the system act as role models and ensure that the formulation and dissemination of priorities and goals are facilitated in a manner that targets everyone.^7^ The PHC conceptual framework for monitoring and evaluation also assumes that health systems structures and inputs such as governance can lead to improved performance of processes and outcomes. The framework puts good governance as a core component of a resilient healthcare system, where domains such as assessment of the level of political commitment and leadership to PHC is the main vehicle for achieving UHC.^20^ Paradoxically for Malawi, most managers have not been adequately equipped with skills on PMM, as it is not prioritised at the pre-service training level. This seems to underpin the notion possessed by the majority of HCWs that PMM aspects are not core in the delivery of health services. This has consequently disconnected the existing HIS policy, WHO framework for monitoring and evaluation, from the actual practice.

Poor pre-service training is another aspect directly impacting not only knowledge and skills but also the attitudes of most HCWs, who seem to be unconcerned with the tasks and the benefits of performance measurement. It is acknowledged that appropriate and adequate training is vital for enhancing competence in HCWs, and for delivery of quality health services, which can be done through experience, pre- and in-service training, and mentorship.^21^ Training interventions such as in-service and pre-service training are key in effecting processes of PMM.^22^ It is further recognised that performance improvement is nurtured through medical training institutions for Malawi who would include issues of PMM in their curriculums. We have seen that these have not been adequately utilised to drive the performance culture among HCWs to the expected standards. The experience then is that HCWs either have huge workload or have not been well prepared in their pre-service training to value and handle PMM which, further prevents them from prioritising the data management tasks since efforts. The HCWs are then, by default more comfortable with routine, protocolised clinical services than in critical sensemaking of what they do and how to improve based on the information they gather in their daily practice.

Also, Malawi’s PMM is characterised by financial constraints within the PHC system which are crippling the PMM tasks. Availability of financial resources could have perfected the PMM practices through in-service, mentoring, and coaching, thereby providing a vital perspective that ‘not all’ is lost from inadequate pre-service training. In this case, the training could be used to deal with HCWs’ complacent attitudes towards PMM efforts.^22^ The effects of financial challenges are also carried over to impact on the processes of generating and using performance information. Though the goal of HIS policy is to ensure that information generated is timely, accurate and user-friendly for the purposes of monitoring, evaluating, and improving healthcare services, the current status quo makes this difficult. Again, the financial and material challenges in the health system have hindered not only employment of more qualified data officers, but also the capacity building processes for HCWs in PMM. This has posed a threat to the timeliness, completeness and accuracy of information generated. The lack of quality information is also seen as one aspect hampering its utilisation.^23^ No wonder our findings show that most decision-makers make little use of data because they question its trustworthiness. It is recognised that relevant, accurate, quantitative and qualitative data that are collected and used in a timely and efficient manner are essential for delivery of patient/consumer care and management of services.^24^ Accurate public health information is essential for monitoring and evaluating health and for improving the delivery of healthcare services and programmes.^25^

On the other hand, the finding that well-funded programmes are performing better consolidate the fact that financial resources are a significant contributor to PMM not only for PHC but other levels of health care. Among the performance tools adopted by such well-funded programmes are appropriate supervision and performance feedback. Supervision is described as not only an important lever in attaining an organisational goal but also a tool for quality assurance.^26^ Feedback, on the other hand, is aimed to improve data quality and decision-making processes. It is viewed as a strategy for correcting errors and strengthening of the health management aspects such as problem identification at group and organisational levels as a step towards performance improvement.^27^

It has been noticed that some HCWs feel uncomfortable with the supervisors from the top level, who are largely involved in supervising and providing feedback to the HCWs. It appears this is because of authoritative nature of the supervisory processes. It is recommended that supervision and feedback meetings should be informal and relaxed to serve its purpose.^28^ This study finds it imperative that PMM should move away from being reactionary and top-down to being a routine collegial practice with adequate accountability guardrails both vertically and horizontally; including the end users. While we acknowledge the lack of time and resources as a deterrent, changing the manner in which it happens is pivotal.^29^ For instance, there is a need for supervision to be supportive and continuous in order to eliminate the aspects thwarting the PMM processes.

Available evidence also shows that the use and non-use of incentives and rewards to drive HCW performance.^30^ Though the notion of the use of financial resources as a performance tool appears to be skirted, its impact cannot be underestimated in the present world, as the needs and aspirations of people continue to increase. In Malawi, for instance, it was found that financial incentives in the form of salaries, training or meetings allowances, bonuses, and refresher courses are among key motivators for Health Surveillance Assistants (HSAs). Training was also found to motivate most HSAs since it was seen as a means of sourcing income through allowances. Other motives for training were career progression, mentorship, and capacity building.^31^ For Malawi, the use of rewards for PM appears to be wanting. Intrinsic and extrinsic motivation for the implementation of PMM is still a big challenge. As highlighted, the lack of motivation towards PMM is emanating from various aspects such as deficiency of financial resources, and problems associated with leadership, compounded by a lack of understanding on the process, use, and importance of performance information by the majority of HCWs. This shows the need to change the current status quo by navigating to a system which can balance the use of both intrinsic versus extrinsic and, monetary versus non-monetary motivational mechanisms.

We acknowledge that Malawi’s health system is heavily subsidised by donor support, whose parallel incentivisation system is often monetary.^31^ We need motivational approaches that are manageable and sustainable within the PHC organisations to drive PMM functions. Already, it is encouraging to note that some facility in-charge are innovative in the explorations and use of non-monetary motivations such as verbal appraisals and delegation of responsibility to institutionalise the PMM culture. While vertical programmes are thriving on financial incentives, the authors however acknowledge that it is imperative to also explore means of overcoming the financial burden so that the PMM processes become effective and sustainable. This study hence recommends innovations that foster a good balance between intrinsic and extrinsic motivational measures to PMM. How we can leverage on the lessons and gains from vertical programmes and yet remain sustainable is a question for future research.

### Limitations

Firstly, data for this study were collected from 5 districts of Malawi, and 15 HCs. The data obtained therefore may not give a generalisable characteristic of PMM practices for Malawi. However, we tried to mitigate this by sampling one district in each of the health zones. Secondly, this study relied on the information which participants provided mostly based on their views and observations. A mixed method could be appropriate to triangulate the IDIs with observation method or document analysis. Also, the likelihood that participants could not be honest was there, since they were directly involved in PMM tasks. The last limitation was that the investigators, given the fact that they have experiences in how health system operates in Malawi, might have brought their experiences and feelings into the study. However, this was minimised by engaging outside people in the formulation of data collection tools, data coding, and the writing of summaries of the findings.

## Conclusion and recommendations

Malawi, just like many LMICs, needs support for enablers of PMM in improving the PMM practices, for the improvement of processes, and outputs of PHC. There is an urgent need therefore to improve not only the capacity to supply more human and material resources for PMM, but also institute a better strategy for retaining HCWs within the system. For instance, the system could enhance the induction of the novice HCWs and offer extra incentives to the ones working in the hard to reach areas. The system needs to strengthen the utilisation of continuous professional development (CPD) in form of audits and reviews in formalised and non-formalised ways. The use of CPD may not require a lot or any financial resources as workers can always collaborate to assist each other on various areas within the workplace. The system can also effectively use feedback and supervision as tools to attain the set goals. To do this, there is need to capacitate managers at every level with leadership skills. The skills will help leaders to institute a positive working culture that is well aligned with the system’s goals and priorities. There is also a need to lobby for more funding in the health sector for the enhancement of both pre- and post-service training to incorporate aspects of PMM as core aspects to improvement of leadership and management at all levels of PHC. There is also the need for further studies on innovations that enhance the rewarding, negative sanctioning and accountability systems within PHC in Malawi. This can be done by creating mandatory policies on how HCWs can be rewarded or sanctioned in relation to PMM.
